# Transcriptomics integrated with metabolomics reveals the effect of cold stress on rice microspores

**DOI:** 10.1186/s12870-023-04530-2

**Published:** 2023-10-27

**Authors:** Yingbo Li, Yingjie Zong, Wenrui Li, Guimei Guo, Longhua Zhou, Hongwei Xu, Runhong Gao, Chenghong Liu

**Affiliations:** 1 Biotech Research Institute, Shanghai Academy of Agricultural Sciences, Shanghai, China; 2 Key Laboratory of Agricultural Genetics and Breeding, Shanghai, China

**Keywords:** Microspore embryogenesis, Cold stress, Rice, Transcriptome, Metabolomics

## Abstract

**Background:**

Microspore culture is one of the important biotechnological tools in plant breeding. The induction of microspore embryogenesis is a critical factor that affects the yield of microspore-derived embryo productions. Cold treatment has been reported to reprogram the gametophytic pathway in various plant species. However, the exact mechanism(s) underlying the effect of cold pre-treatment of floral buds on the efficiency of ME is still not clear.

**Results:**

In this study, the effects of cold stress on the microspore totipotency of rice cultivar Zhonghua 11 were investigated. Our results revealed that a 10-day cold treatment is necessary for microspore embryogenesis initiation. During this period, the survival rate of microspores increased and reached a peak at 7 days post treatment (dpt), before decreasing at 10 dpt. RNA-seq analysis showed that the number of DEGs increased from 3 dpt to 10 dpt, with more downregulated DEGs than upregulated ones at the same time point. GO enrichment analysis showed a shift from ‘Response to abiotic stimulus’ at 3 dpt to ‘Metabolic process’ at 7 and 10 dpt, with the most significant category in the cellular component being ‘cell wall’. KEGG analysis of the pathways revealed changes during cold treatment. Mass spectrometry was used to evaluate the variations in metabolites at 10 dpt compared to 0 dpt, with more downregulated DEMs being determined in both GC-MS and LC-MS modes. These DEMs were classified into 11 categories, Most of the DEMs belonged to ‘lipids and lipid-like molecules’. KEGG analysis of DEMs indicates pathways related to amino acid and nucleotide metabolism being upregulated and those related to carbohydrate metabolism being downregulated. An integration analysis of transcriptomics and metabolomics showed that most pathways belonged to ‘Amino acid metabolism’ and ‘Carbohydrate metabolism’. Four DEMs were identified in the interaction network, with stearidonic acid involving in the most correlations, suggesting the potential role in microspore totipotency.

**Conclusions:**

Our findings exhibited the molecular events occurring during stress-induced rice microspore. Pathways related to ‘Amino acid metabolism’ and ‘Carbohydrate metabolism’ may play important roles in rice microspore totipotency. Stearidonic acid was identified, which may participate in the initiation of microspore embryogenesis.

**Supplementary Information:**

The online version contains supplementary material available at 10.1186/s12870-023-04530-2.

## Background

Doubled haploids (DHs) are important biotechnological tools in plant breeding [1]. Microspore culture is one of the most common tools for DHs based on cell totipotency in plants. Under the application of stress treatment, the single haploid cell microspores are induced into callus in vitro [2]. The Microspore-derived embryo callus (MDEC) can be diploidized spontaneously or in response to chemical agents, finally producing doubled haploid plants [3]. During Microspore embryogenesis, the variation can be easily fixed into a complete homozygous state. To date, numerous reports on microspore embryogenesis have been reported in crop plants. However, the yield of MDEC is still one of the major factors that limit the acquisition of DH plants.

The induction of embryogenesis is a critical factor that affects the yield of microspore-derived embryo productions [4]. It has been demonstrated that specific stress treatments are necessary for the successful induction of microspores to switch to the embryogenic pattern of development, leading to the formation of haploid embryos [5, 6]. Cold treatment has been reported to reprogram the gametophytic pathway in various plant species. Spike cooling is a commonly used preculture stress in barley microspore cultures [7, 8]. In Brassica, exposure of harvested buds to cold conditions for a few days is essential for the androgenic switch and can enhance the efficiency of microspore embryogenesis [9]. Because of the lack of clear cytological markers to identify microspore cells that undergoing reprogramming, bioinformatic analyses are useful tools to investigate the molecular mechanism during the induction stage. Transcriptomic analysis revealed that genes encoding heat shock proteins and cytochrome P450s were upregulated to maintain cellular homeostasis and reduce oxidative damage in soybean microspores under cold stress [10]. Despite the progress made in recent years, the exact mechanism(s) underlying the effect of cold pre-treatment of floral buds on the efficiency of ME is still not clear. Therefore, a comprehensive understanding of the mechanisms that induce the sporophytic pathway under cold stress would be beneficial in optimizing culture conditions and improving the yield of embryoids.

Rice is one of the most important staple food crops, as it supports more than half of the world’s population. There are numerous reports in rice anther culture, and the reports on haploid production via isolated microspore culture are relatively scarce. DATTA et al. [11] reported a system of microspore culture in both indica and japonica rice. Xie et al. [12] showed maltose and growth regulator combination can improve microspore culture efficiency in japonica rice. Chowdhury and Mandal harvest regeneration plants by microspore culture using salt-susceptible and tolerant rice hybrid [13]. In recent reports, Rahman et al. [14] established microspore culture protocol in Malaysian indica rice MR219. In our previous study, we successful harvest plants with microspore culture in japonica rice Zhonghua 11. However, the production of MDEC was still lower in rice compared with that in Barley and Brassica. Hence, research on the molecular changes during rice microspore induction is crucial to improving the callus induction efficiency.

In the present study, we investigated the change of microspore activity during cold treatment, and determined the shortest cold temperature treatment time in rice microspore-inducing callus. We further integrated transcriptome and non-targeted metabolome to clarify the mechanism of the effect of cold stress. The hope is that the effect of cold stress can be explained from an integrative omics perspective and provide a theoretical basis and guidance for the microspore inducing-callus of rice and monocot crops.

## Result

### Influence of cold stress on the rice MDEC induction

**Fig. 1:**
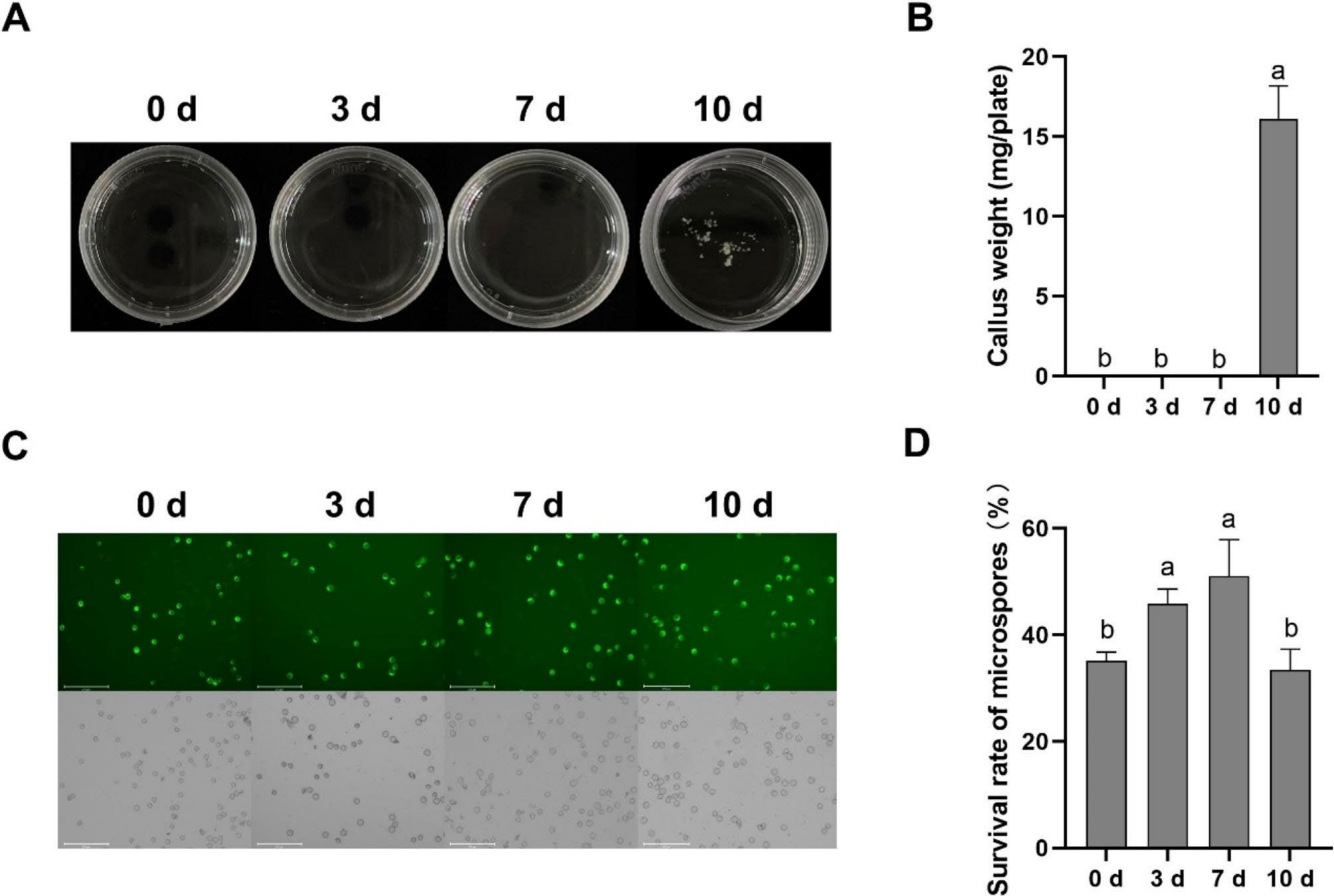
(**A**) In vitro culture of Zhonghua 11 MDEC at 21 days under different cold treatment time. (**B**) Statistic analysis of Zhonghua 11 MDEC in 21 days culture under different cold treatment time. (**C**) Activity of Zhonghua 11 microspores by FDA stain under different cold treatment time. (**D**) Statistic analysis of the survival rate in Zhonghua 11 microspores under different cold treatment time. ‘*’ means p < 0.05. Bars = 275 μm

During the induction of rice MDEC, the harvested spikes were exposed to cold conditions for several days to determine the exact treatment time. Three time intervals (3, 7, and 10 days) were set in the experiments, with 0 days as the control. It was found that when treated with 10 days of cold conditions, the rice microspores could be induced into calli, while none were gained with less than 10 days’ cold treatment (Fig. 1A and b). This indicated that 10 days of cold treatment is essential for ZhongHua 11 MDEC formation.

**Fig. 2:**
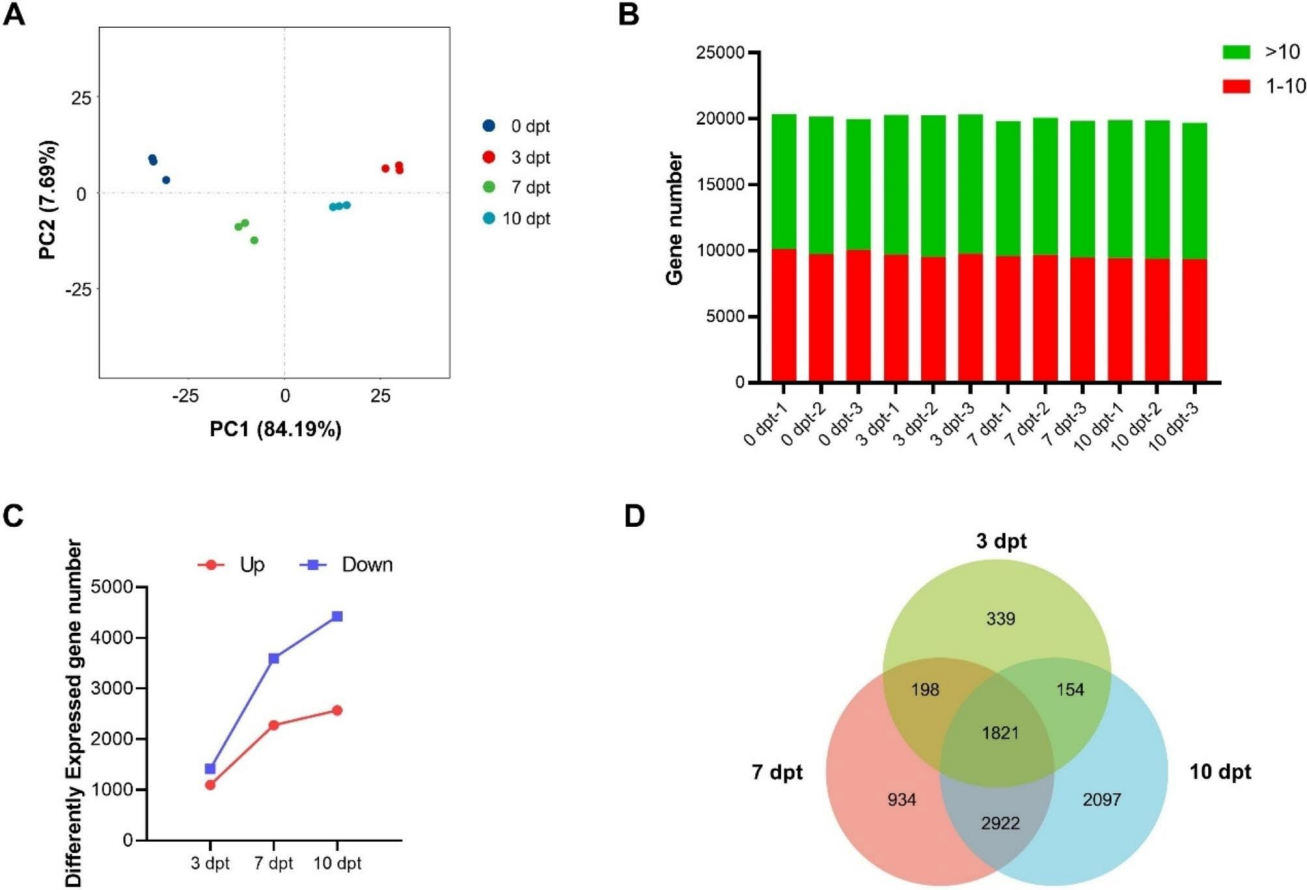
Comparative analysis of transcripts in Zhonghua 11 microspores under cold treatment. (**A**) PCA analysis of the samples from the four time points. (**B**) Whole genomic gene expression at 0, 3, 7, and 10 dpt. The y axis represents gene number. Red box means fragments per kilo bases per million reads (FPKM) 1–10, green box means FPKM > 10. Number of differently expressed genes in at three time points (3, 7, and 10 dpt). The y-axis represents DEGs number. (**C**) Number of differently expressed genes in Zhonghua 11 microspores at three time points (3, 7, and 10 dpt) compared with 0 dpt. The **y** axis represents DEGs number. Number of DEGs was counted with the criteria *p* < 0.05 and log_2_ (fold change) ≧ 1. Red line shows up-regulated DEGs, blue line shows down-regulated DEGs. (**D**) Venn diagram comparison of DEGs at three cold treatment time points (3, 7 and 10 dpt) in Zhonghua 11 microspores

The survival percentage of microspores was further analyzed at each treatment time point (Fig. 2A and B). As shown in Fig. 2B, the survival percentage of microspores increased from 0 to 7 dpt (days post-treatment) gradually but decreased at 10 dpt. The survival percentage of microspores at 10 dpt had no significant difference with that at 0 dpt. This suggested that the formation of MDEC was dependent on long-term cold stress, but the survival percentage of microspores had no effect on the formation of MDEC. Transcriptomics and metabolomics analysis were conducted to further investigate the molecular changes in rice microspores under long-term cold stress.

### RNA sequencing and data analysis of the rice microspores under cold stress

RNA-seq was performed on the rice micropores with none cold treatment (0 dpt) and longtime cold treatment (3, 7, and 10 dpt). The cDNA libraries were constructed from 12 samples separately and sequenced on the Illumina sequencing platform. In average, 49.21 million raw reads were generated from each library (Supplemental table S1). After removing the low-quality reads, the total numbers of remained reads per library ranged from 46.07 to 49.83 million. The ‘*Phred* value’ > 30 (Q30) of each library ranged from 91.35 to 91.76%. And the GC percentage of each library ranged from 53.56 to 54.28%. PCA analysis of the gene expression profiles indicated that the first (PC1) and second (PC2) principal components accounted for 84.19% and 7.69% of the total variance, respectively. The results showed that the samples obtained from the four time points were divided into four distinct regions (Fig. 2A). The transcriptome data of all samples was qualified to be used for further analysis of differentially expressed genes (DEGs).

Whole genomic gene expression at four time points was compared. Analysis results showed the average expressional intensity (FPKM ≥ 1) of whole genomic genes expressed at the highest level at 3 dpt than that at other time points (Fig. 2B). Differentially expressed genes (DEGs) were counted using transcripts at 3, 7, and 10 dpt [0 dpt was used as control, p < 0.05, and log_2_(fold change ≧ 1)], respectively. The expressional pattern of DEGs was displayed as the upregulated group and the downregulated group (Fig. 2C). Number of the DEGs in both groups increased from 3 dpt to 10 dpt. The number of DEGs in the downregulated group was more than that in the upregulated group at the same time. These indicated that more genes were downregulated under long-time cold stress.

Venn diagrams were further constructed using the DEGs counted at three time point (3, 7, and 10 dpt) (Fig. 2D). In summary, 2097 DEGs were specifically detected at 10 dpt, which is higher than the numbers (ranging from 339 to 934) detected specifically at other time points. In total, 1821 DEGs were detected at all three time points. The results suggested that more genes were regulated and functioned during cold stress.

### Validation of RNA-seq results by quantitative RT‑PCR

**Fig. 3:**
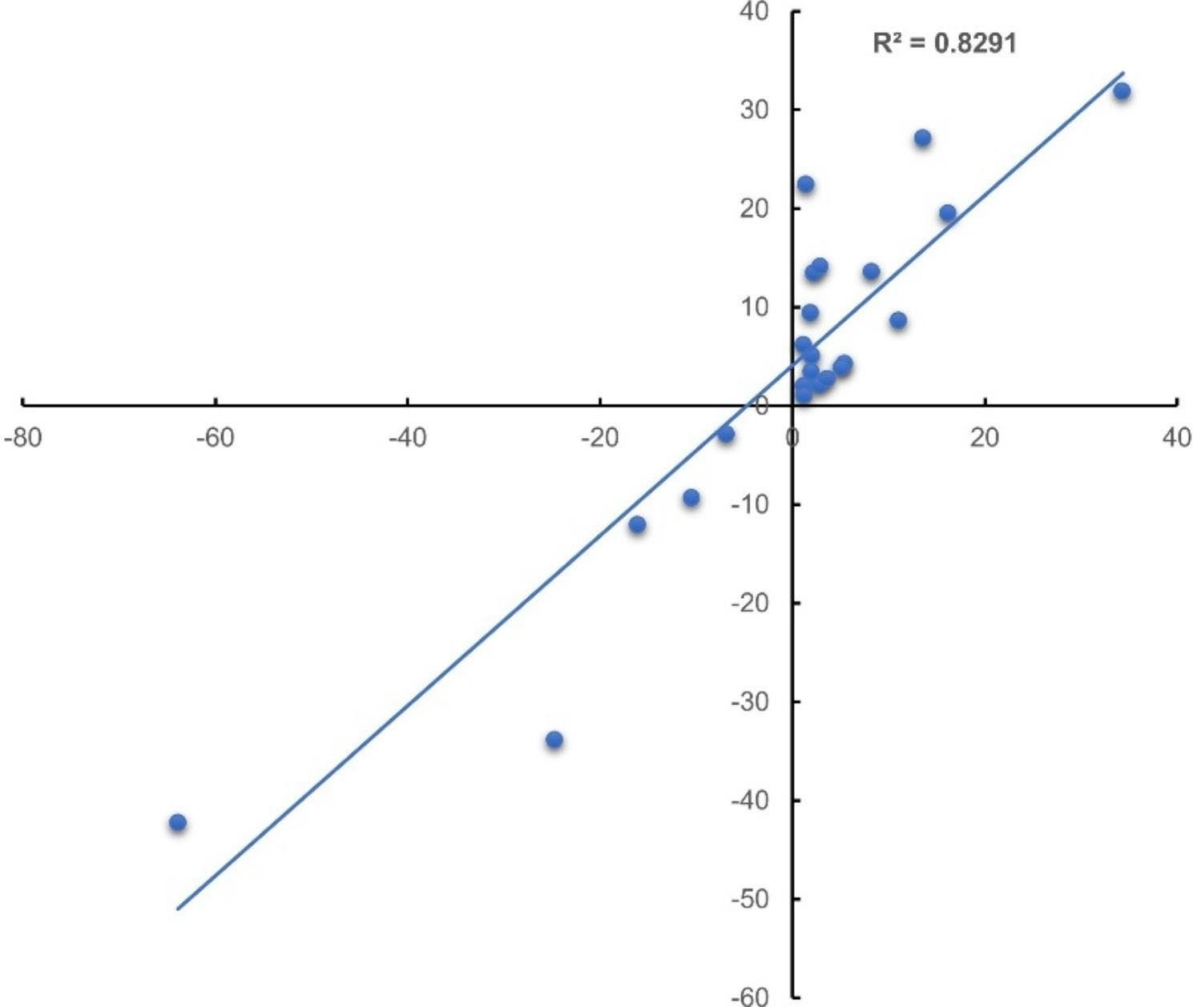
Validation of transcriptome data by qRT-PCR. Scatter plots indicate the transcriptional changes of qRT-PCR analysis and RNA-seq for 22 data points from 10 genes in 4 samples. The Pearson correlation coefficient (R^2^) was 0.8291

To validate the results of the gene expression from RNA-seq data, 10 DEGs were selected for qRT-PCR analysis (Supplemental table S2). There was a good concordance (R^2^ = 0.8291) between RNA-seq data and qRT-PCR analysis (Fig. 3), indicating that the gene expression levels by DGE analysis were reliable.

### Expression pattern of transcripts during cold stress

**Fig. 4:**
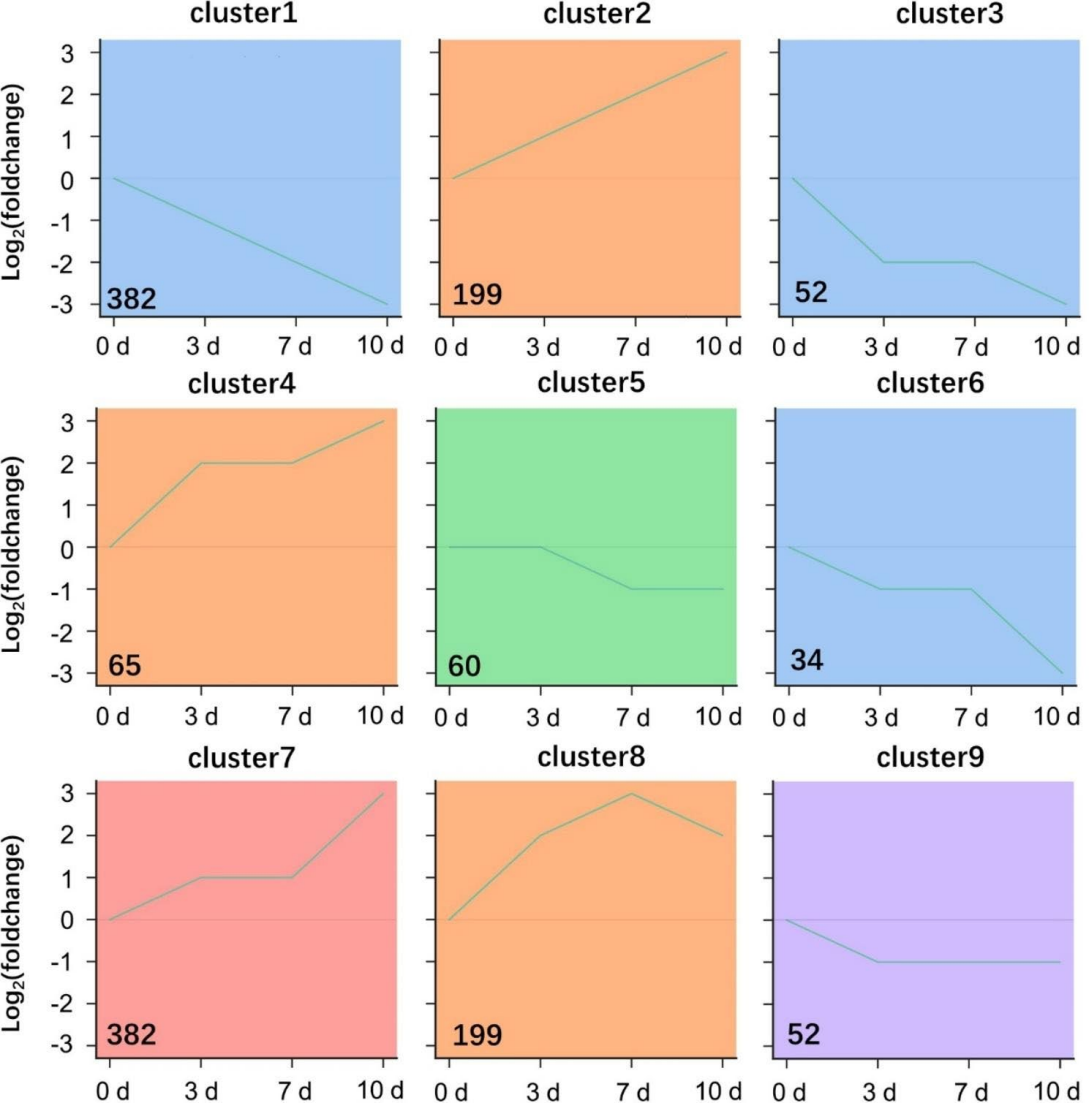
STEM clustering of transcript expression profiles over cold treat time-course. Prior to cluster analysis, 0 dpt was set as control, and the data were standardized. Each diagram shows the relative transcript levels at 3, 7, and 10 dpt, respectively. The number of transcripts is given for each cluster

To group the transcripts based on the expression patterns during cold stress. Stem clustering was performed on all transcripts (FPKM ≥ 1) by time course. Nine clusters were grouped under the criteria (*p* < 0.05) (Fig. 4). The clusters can divide into decreased and increased patterns. Clusters 1, 3, 5, 6, and 9 showed a decreased expression pattern in response to cold stress. In this expression pattern, genes in cluster 9 downregulated from 0 to 3 dpt and were unchanged in the subsequent time points. Genes in cluster 5 downregulated from 3 to 7 dpt and were unchanged in the subsequent time points. Clusters 2, 4, 7, and 8 showed increased expression patterns. The expression pattern of genes in cluster 8 reached a peak at 7 dpt and decreased in the following time point.

### GO and KEGG enrichment analysis of DEGs under cold stress

**Fig. 5:**
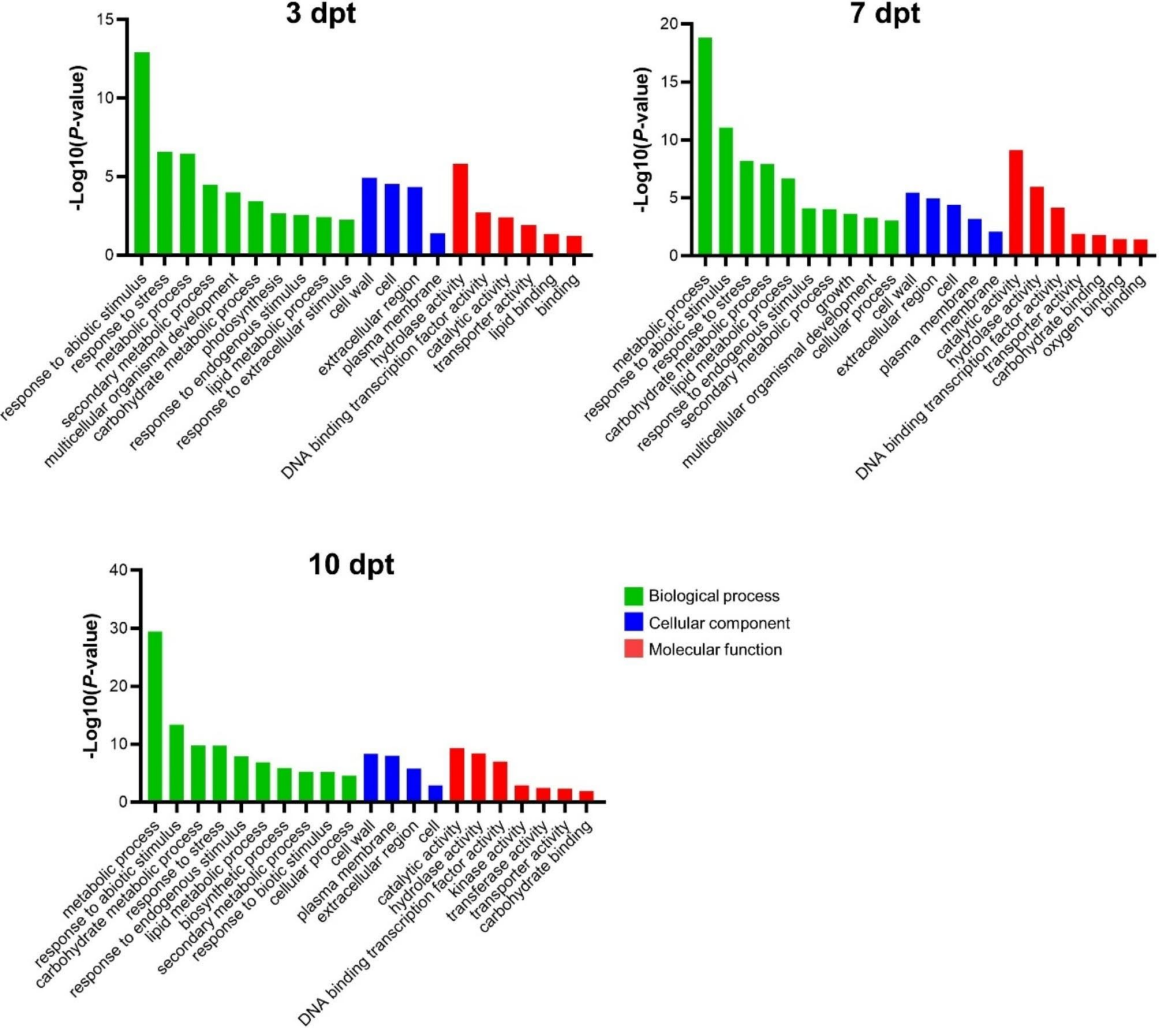
GO analysis of DEGs at three cold treatment time point with the criteria *p* < 0.05

Gene Ontology (GO) functional analysis was deployed by assigning all DEGs at three time points to evaluate the main biological processes under cold stress, respectively (Fig. 5). The results showed response to stress (abiotic, endogenous) and metabolic process (secondary, carbohydrate, lipid) were most significant in biological process. The categories from 7 to 10 dpt were similar. ‘Response to abiotic stimulus’ was the most significant category in biological process at 3 dpt. ‘Metabolic process’ was the most significant category in biological process at 7 and 10 dpt. ‘Cell wall’ was the most significant category in cellular component at three time points. ‘Hydrolase activity’ was the most significant category in molecular function at 3 dpt. At 7 and 10 dpt, the most significant molecular function was ‘catalytic activity’. These suggested the longtime cold stress (7 to 10 days) induced adaptation mechanism to environmental changes in microspore cells.

KEGG enrichment analysis of different biological pathways in microspores during cold stress was conducted using DEGs from three time points (Table 1). Results showed that the ‘Protein processing in endoplasmic reticulum’ pathway was upregulated from 3 to 10 dpt, while ‘Photosynthesis-antenna proteins’, ‘Zeatin biosynthesis’, ‘Alpha-Linolenic acid metabolism’, ‘Plant-pathogen interaction’, ‘Nitrogen metabolism’ and ‘Glutathione metabolism’ were downregulated pathways. ‘Glycerolipid metabolism’ was the only upregulated pathway from 7 to 10 dpt. In contrast ‘Glycolysis/Gluconeogenesis’, ‘Pentose phosphate pathway’, ‘Fatty acid elongation’, ‘Pyruvate metabolism’, ‘Diterpenoid biosynthesis’, ‘Fatty acid biosynthesis’, and ‘Fructose and mannose metabolism’ were downregulated pathways. Eight pathways were especially enriched at 10 dpt. ‘Valine, leucine and isoleucine degradation’, ‘Amino sugar and nucleotide sugar metabolism’, and ‘Porphyrin metabolism’ were upregulated pathways, while ‘Cysteine and methionine metabolism’, ‘Sulfur metabolism’, ‘Phenylalanine, tyrosine and tryptophan biosynthesis’, ‘Thiamine metabolism’, and ‘Biotin metabolism’ were downregulated. Based on the above microspore culturing experiment, these pathways may be important for microspore totipotency.

**Table 1:** DEGs enriched in the pathways at 3, 7, and 10 dpt. ≥1means upregulated gene numbers, ≤−1 means downregulated gene numbers

Pathways	3 dpt		7 dpt		10 dpt	
	≥ 1	≤−1	≥ 1	≤−1	≥ 1	≤−1
Protein processing in endoplasmic reticulum	23		37		45	
Starch and sucrose metabolism	12	15	21	36	31	
Cutin, suberine and wax biosynthesis	5	6		15		18
Galactose metabolism	5				10	
Photosynthesis-antenna proteins		7		9		8
Cyanoamino acid metabolism		10		18		
Phenylpropanoid biosynthesis		21	27	42	36	45
Zeatin biosynthesis		5		10		11
Alpha-Linolenic acid metabolism		7		13		18
Plant-pathogen interaction		17		39		50
Nitrogen metabolism		5		13		16
Glutathione metabolism		9		26		42
Glycerolipid metabolism			10		14	
Glycolysis / Gluconeogenesis			15	28		34
Pentose phosphate pathway				17		18
Fatty acid elongation				14		14
Pyruvate metabolism				20		25
Diterpenoid biosynthesis				11		13
Fatty acid biosynthesis				12		17
Fructose and mannose metabolism				15		17
Valine, leucine and isoleucine degradation					11	
Amino sugar and nucleotide sugar metabolism					22	
Porphyrin metabolism					8	
Cysteine and methionine metabolism						32
Sulfur metabolism						14
Phenylalanine, tyrosine and tryptophan biosynthesis						16
Thiamine metabolism						7
Biotin metabolism						8

### Metabolome analysis of the rice microspores under cold stress

**Fig. 6:**
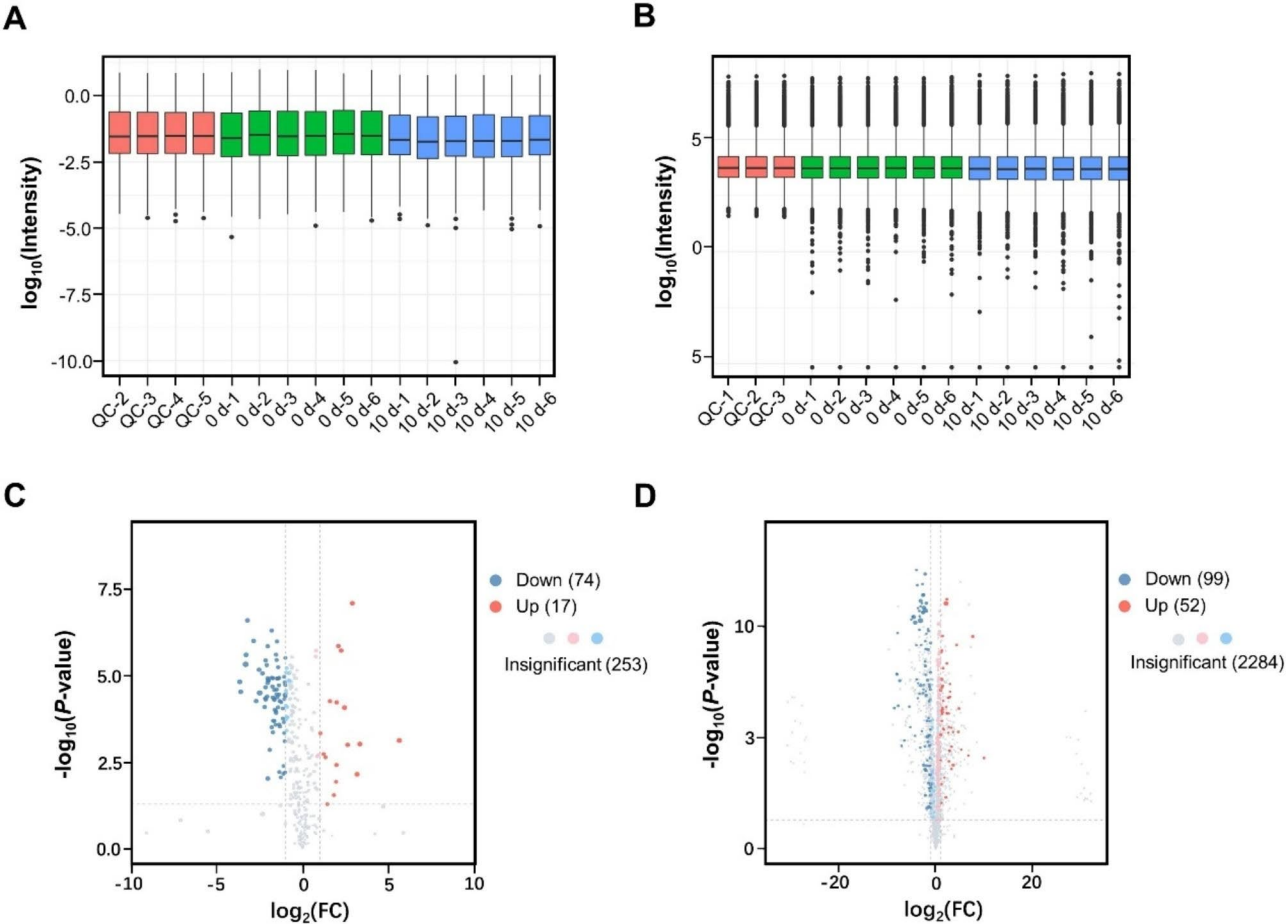
Comparative analysis of metabolites in Zhonghua 11 microspores under cold treatment at two time points (0, and 10 dpt). (**A**) Comparison of whole metabolite expression in GC-MS mode. (**B**) Comparison of whole metabolite expression in LC-MS mode. QC: quality control samples. (**C**) The distribution of DEMs in GC-MS mode. (**D**) The distribution of DEMs in LC-MS mode

**Fig. 7:**
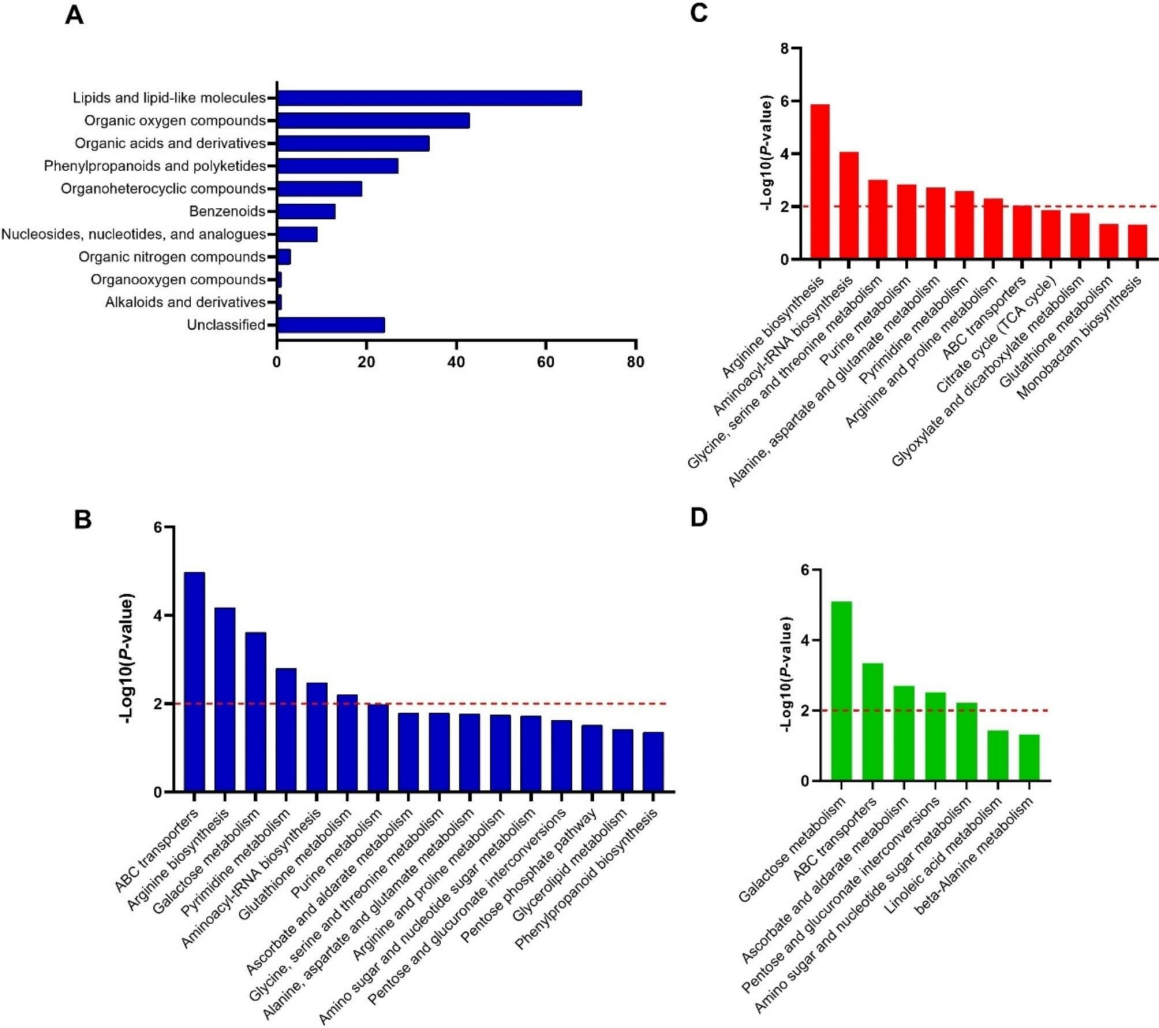
Comparative analysis of DEMs in Zhonghua 11 microspores in both GC-MS and LC-MS modes. (**A**) Classification of DEMs. (**B**) KEGG enrichment analysis of DEMs. (**C**) The enriched upregulated pathways. (**D**) The enriched downregulated pathways. Red dashed line means *p* < 0.01

Because ten days of cold treatment was curial for microspore totipotency, we further determined the metabolite changing between 10 dpt and 0 dpt. GC-MS and LC-MS modes were used in mass spectrometry. The distribution difference of metabolite intensity was low with each group in two modes (Fig. 6A and B). In total, 242 differentially expressed metabolites (DEMs) were identified with the criteria VIP > 1 and |log_2_ FoldChange| ≥ 1.0 by combining of the two modes. The number of DEMs identified using the GC-MS was 91. Among them 74 were downregulated and 17 were upregulated (Fig. 6C). 151 DEMs were identified by using the LC-MS, including 99 downregulated and 52 upregulated DEMs (Fig. 6D). The proportion of metabolites according to their chemical taxonomy was analyzed. All DEMs can be classified into 11 categories (Fig. 7A). A higher number of DEMs belonged to the ‘lipids and lipid-like molecules’, ‘Organic oxygen compounds’, ‘Organic acids and derivatives’ and ‘Phenylpropanoids and polyketides’ categories.

KEGG enrichment analysis was performed using the DEMs. Sixteen pathways (p < 0.05) were identified in the KEGG enrichment analysis. The most significant (p < 0.01) enriched KEGG terms were ‘ABC transporters’, ‘Arginine biosynthesis’, ‘Galactose metabolism’, ‘Pyrimidine metabolism’, ‘Aminoacyl-tRNA biosynthesis’, ‘Glutathione metabolism’ (Fig. 7B). In the upregulated group, the most significant (p < 0.01) KEGG terms were ‘Arginine biosynthesis’, ‘Aminoacyl-tRNA biosynthesis’, ‘Glycine, serine and threonine metabolism’, ‘Purine metabolism’, ‘Alanine, aspartate and glutamate metabolism’, ‘Pyrimidine metabolism’, ‘Arginine and proline metabolism’ and ‘ABC transporters’ (Fig. 7C). These pathways belong to ‘Amino acid metabolism’, ‘Nucleotide metabolism’, and ‘Metabolism of cofactors and vitamins’. The most significant (p < 0.01) enriched KEGG terms in the downregulated group were ‘Galactose metabolism’, ‘ABC transporters’, ‘Ascorbate and aldarate metabolism’, ‘Pentose and glucuronate interconversions’, and ‘Amino sugar and nucleotide sugar metabolism’ (Fig. 7D). Most of these pathways belong to ‘Carbohydrate metabolism’.

### Combined transcriptomics and metabolomics analysis

**Fig. 8:**
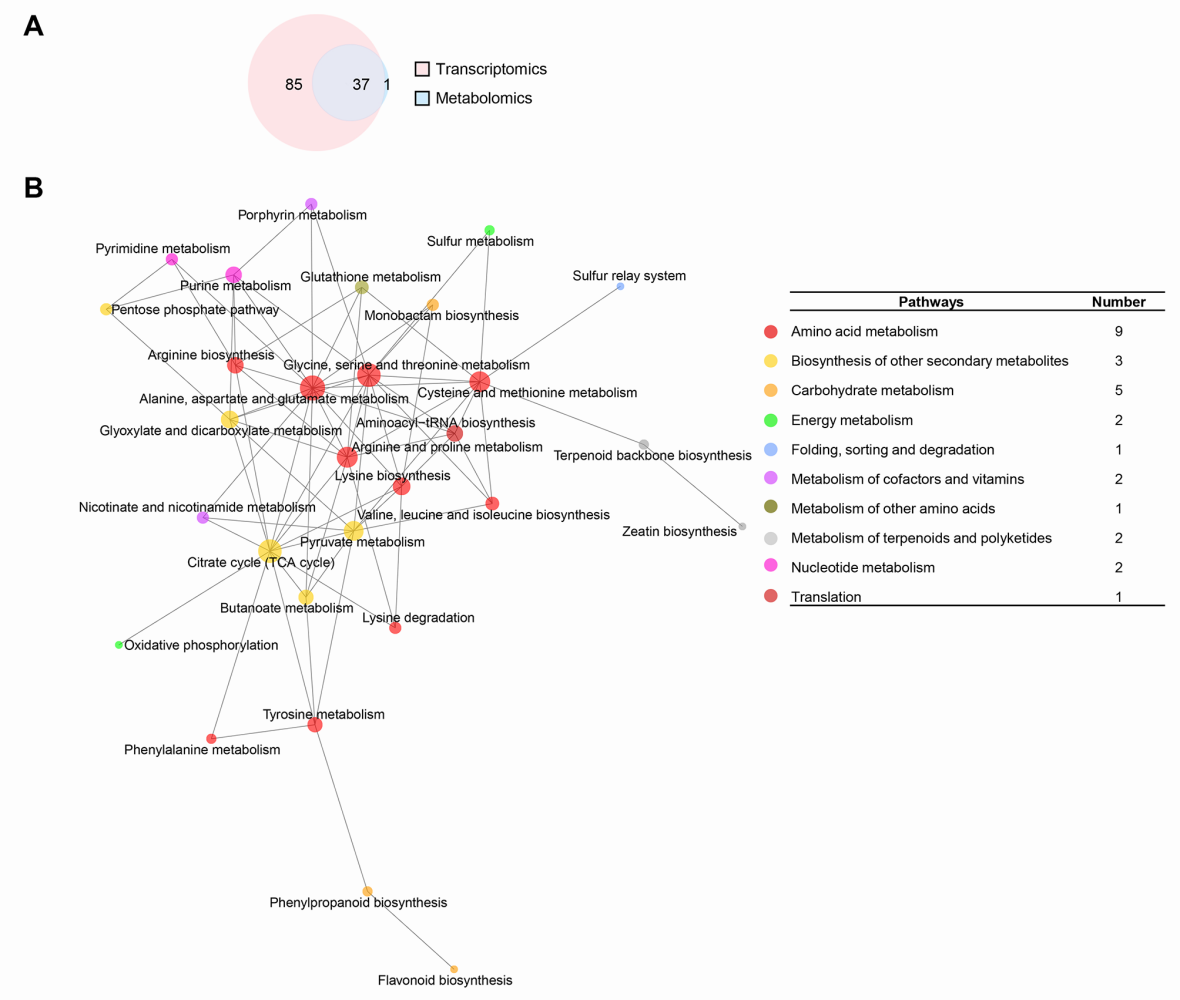
(**A**) Venn diagram comparison of pathways by KEGG enrichment analysis using DEGs and DEMs at 10 dpt. (**B**) The interaction network among pathways in both transcriptomics and metabolomics. Different colored circles mean the highest-level pathways. The number of next level pathways is given behind the highest-level pathways

To determine the association between DEGs and DEMs under cold stress, a combined analysis of transcriptome and metabolome data was carried out using KEGG database. The DEGs and DEMs from 10 dpt were mapped to the related KEGG metabolic pathways. A Venn diagram was constructed between DEGs and DEMs related pathways. Thirty-seven pathways were detected in both groups (Fig. 8A). Interaction network diagram among the 37 pathways was drawn (Fig. 8B). The results showed that pathways belonging to ‘Amino acid metabolism’ and ‘Carbohydrate metabolism’ were most common (Fig. 8B, right). This suggested these two types of pathways may play important roles in microspores under cold stress.

**Fig. 9:**
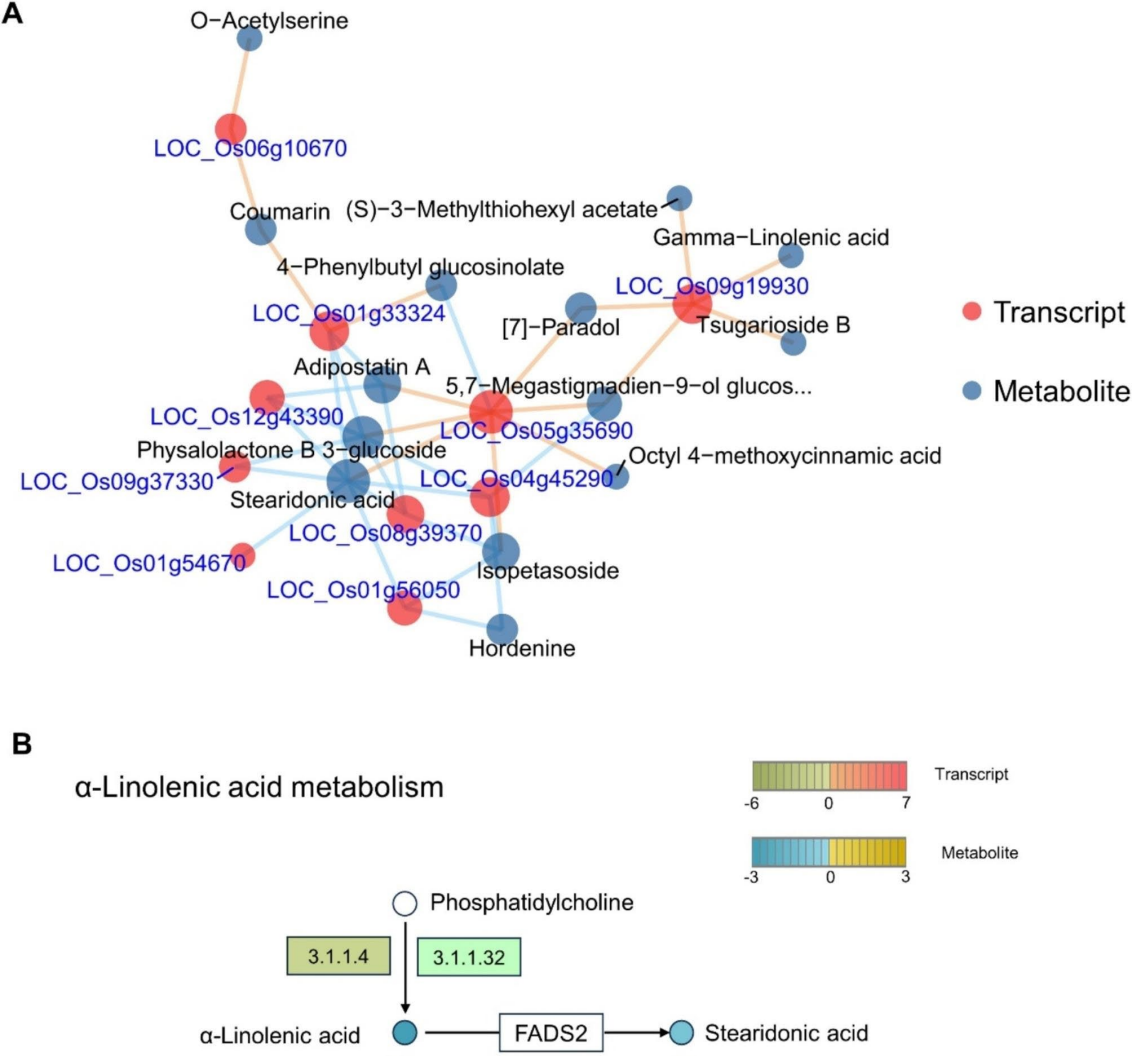
(**A**) The interaction network among genes and metabolite. The light-yellow line means positive correlation, the light blue line represents negative correlation. (**B**) Changes in Stearidonic acid in Zhonghua 11 microspores under cold stress

Pearson correlation algorithm was used to compute the relationship between candidate metabolites and genes, and a network diagram of candidate metabolites and genes was drawn (Supplemental Fig. S1). In the network diagram, the more associated it is with other indicates the greater impact. A network with the most correlations was further peaked (Fig. 9A). The DEMs were selected under the strategy with clear KEGG pathway annotations. This strategy may have missed some interesting DEGs and DEMs, but this was counterbalanced by the reliability of digging targeted genes specific to the microspore totipotency. Four DEMs were selected, including O-Acetylserine, Adipostatin A, Stearidonic acid, and Coumarin. Stearidonic acid with the most correlations among the four DEMs (Supplemental data 1). Stearidonic acid was downregulated at 10 dpt (Fig. 9B). This suggested the downregulation of stearidonic acid may play an important role in the initiation of microspore embryogenesis.

## Discussion

Cold stress treatment was the necessary condition for the induction of microspores. In our study, the japonica rice cultivar Zhonghua 11 needs 10 days of cold treatment to initiate the MDEC induction. This was in consistent to the earlier reports, which all showed the rice MDEC induction requires cold treatment [12–14]. However, the cold treatment conditions in our study were different from the previous reports. Xie et al. [12] recommended the japonica rice panicles were cooled at 6 °C for 15–27 days. The treatment condition was 8 °C for 7–10 days [13] and 8 °C for 7 days [14] in the indica rice cultivar. The difference in cold treatment conditions may be due to the different rice cultivars we used in the present study.

Several studies have shown a marked increase in cell death levels after stress treatment during microspore embryogenesis [15]. Prem et al. [9] showed the existence of a high proportion of dead cells in the rapeseed microspore culture. Bárány et al. [16] showed the similar results in barley. The microspores they determined in these two studies were isolated and cultured for several days under cold stress. This is different from ours, which were immediately isolated after cold treatment and determined. Rodríguez-Serrano et al. [17] showed the proportion of dead cells increased after 23 days of cold treatment in barley microspore and suspension cultures. In the present study, there is no significant difference in the survival percentage of rice microspores at 10 dpt compares with that at 0 dpt. The difference is probably due to the too-long-time cold treatment in barley spikes. Moreover, a four days low temperature treatment supplemented with nitrogen/carbohydrate starvation stress increased the triticale microspore viability in vitro culture [18]. This is similar with our results which showed the survival percentage of microspores was increased from 0 to 3 dpt. Because the survival percentage of microspores decreased from 7 to 10 dpt in our results, these also indicated that the appropriate duration of cold treatment is important for the yield of MDEC.

The average expressional intensity of whole genomic genes increased from 0 to 3 dpt, and decreased slightly in the following time points. Seifert et al. [19] the number of genes expressed in microspores increased under 10 days cold treatment. Bélanger et al. [20] showed similar results in barley microspores under stress treatment. This indicated a common response among plant microspores under treatment.

Our results showed that the number of DEGs increased during cold treatment, and downregulated genes were more than upregulated at the same time. It is well known that low temperatures can reduce cell viability. GO analysis also indicated that the DEGs were enriched in metabolic and response to stress biological processes. Interestingly, the significant biological progress switched from ‘Response to abiotic stimulus’ at 3 dpt to ‘Metabolic process’ at 7–10 dpt. This indicates the microspore adapt to the low temperature from 7 to 10 days. Plant cell walls were reported play important roles in the regulating plant growth, development, and determining of cell shape and fate [21]. Cell wall remodeling was required in cell division and expansion [3]. Bárány et al. [22] showed the different distribution patterns of cell wall polymers are markers of proliferation and differentiation events in pollen reprogramming to embryogenesis. In our results, ‘cell wall’ was the most significant cellular component among DEGs from 3 to 10 pt. This suggested that cold stress plays a curial role in rice microspore totipotency by inducing the cell wall changes.

It is hypothesized that the decreased biosynthesis of starch and lipid bodies may play an important role in microspore totipotency [23]. In our results, KEGG analysis showed genes belonging to the ‘Fatty acid elongation’ and ‘Fatty acid biosynthesis’ were downregulated from 7 to 10 dpt. More downregulated genes that enriched in ‘Starch and sucrose metabolism’ pathway than upregulated genes from 3 to 7 dpt. However, more upregulated genes were detected in ‘Starch and sucrose metabolism’ pathway at 10 dpt. In the previous study, researchers determined the gene expression change in microspores following mannitol treatment [24]. This is different from ours which used spikes following cold treatment. Shishova et al. [25] showed that sugars exert energy providing and signal transducing functions in pollen development. Hale et al. [10] showed that cold stress enhanced the biosynthesis of carbohydrate derivatives, including those produced by nonhydroxyl sugar modification. These suggested the development of microspores needs ‘Starch and sucrose metabolism’ pathway to provide energy. Moreover, four pathways (‘Glycolysis/Gluconeogenesis’, ‘Pentose phosphate pathway’, ‘Pyruvate metabolism’, and ‘Fructose and mannose metabolism’) related to ‘Carbohydrate metabolism’ were downregulated at 7 and 10 dpt. These indicated that most of the pathways related to ‘Carbohydrate metabolism’ were downregulated. These in consistent with the previous hypothesis.

More downregulated DEMs were determined in both GC-MS and LC-MS modes. This in consistent with the transcriptomics that more downregulated DEGs were detected at 10 dpt. Most of the DEMs belonged to ‘lipids and lipid-like molecules’. A hypothesis suggests that the decreased biosynthesis of lipid bodies may play an important role in microspore totipotency [23]. The function of this type of DEMs in microspore totipotency needs further investigation. KEGG analysis of the DEMs showed most of the upregulated pathways were related to ‘amino acid metabolism’, while the downregulated pathways related to ‘Carbohydrate metabolism’. Integration analysis of transcriptomics and metabolomics also showed most of the pathways related to ‘Amino acid metabolism’ and ‘Carbohydrate metabolism’. Kiviharju and Pehu [26] reported the positive effects of cold pre-treatment on callus induction release of substances necessary for androgenesis, mainly amino acids, and shock-thermic proteins. Proline can effectively induce androgenesis in wheat [27]. Hashemi et al. [28] showed proline is one of the most important amino acids to improve microspore culture in eggplant. This suggested that the upregulated pathways related to ‘Amino acid metabolism’ may play important roles in rice microspore totipotency. Under cold treatment, the carbohydrate metabolism was decreased in soybean microspores [10]. In wheat, embryogenesis was efficiently induced by the combination of carbohydrate starvation and heat stress in isolated wheat microspores [29]. this suggested the downregulated pathways related to ‘Carbohydrate metabolism’ may play important roles in the initiation of rice microspore embryogenesis.

The initiation of cell division from stressed microspores has been correlated with a significant decrease in the number and size of lipid bodies [30, 31]. In wheat anther, all of the fatty acids altered during anther development, and the stearic acid levels decreased from the tetrad to late uninucleate stage [32]. These implied fatty acids plant roles in the development of microspores. Stearidonic acid was found with the most correlations in the integration analysis of transcriptomics and metabolomics. This suggested stearidonic acid has correlations with the development of rice microspores, and may play important roles.

## Conclusion

Taken together, in this study we found 10 days cold treatment on spikes is essential for Zhonghua11 microspore embryogenesis. Omics analysis indicates that the initiation of rice microspore embryogenesis induced by cold stress is a complex regulatory network. The upregulated of ‘Amino acid metabolism’ pathways and downregulated of ‘Carbohydrate metabolism’ pathways may play important roles in rice microspore totipotency. Most of the DEMs belonged to ‘lipids and lipid-like molecules’, among which the downregulation of stearidonic acid may play an important role in the initiation of microspore embryogenesis. The function of stearidonic acid in microspore totipotency needs further research.

## Materials and methods

### Plant materials and microspore culture

The experiment was conducted on *japonica* rice cv. Zhonghua 11. The plants were grown at the farm of Shanghai Academy of Agricultural Sciences, Shanghai, China. The stems of the rice varieties containing the developing spikes were harvested. After harvesting, spikes were immediately tightly covered using plastic wrap and aluminum foil, and then incubated at 4 °C for different times (0, 3, 7 and 10 day). Microspores were collected from the spikes under different cold treatment time and preserved in liquid nitrogen for transcriptome (0, 3, 7 and 10 days) and metabolome analysis (0 and 10 days). The remaining microspores were cultured in the induction medium (N6 basal medium supplemented with 2 mg/L 2.4-D, 0.5 g/L L-Glutamine, 0.5 g/L Proline and 60 g/L Sucrose) for embryogenic callus induction. After 21 days of culturing, the formed embryogenic calli was weighed and counted. The experiments have three biological repetitions.

### Survival percentage statistics of microspore

The survival percentage of microspore was analysised by Fluorescein Diacetate (FDA). Microspores were isolated by filtration and centrifugation after different days cold treatment, then stained with 0.01% fluorescein diacetate for 10 min. The microspore activities were counted under fluorescence microscope. Microspores from at least four microscope fields were count in each treatment. More than 50 cells were counted in one microscopic field.

### RNA extraction and preparation of cDNA library

Total RNA was isolated from each microspore sample using Trizol reagent (Invitrogen, Carlsbad, CA, USA) according to the manufacturer’s protocol. RNA integrity was confirmed using the 2100 Bioanalyzer (Agilent Technologies, Palo Alto, CA, USA). RNA libraries can be constructed when 28s/18s of each sample ≥ 0.7 and RIN of each sample ≥ 7. RNA libraries for transcriptome sequencing were constructed according to the IIIumina RNA Seq library kit (Illumina, Inc.). The total RNA was digested by DNase I. Then poly-A containing mRNA was enriched by Oligo (dT) attached magnetic beads, following by random fragmentation of mRNA into small segments. The first and the second strand cDNA were synthesized using the fragments as templates then followed by end repairing. The ends of DNA fragments were modified and ligated with adapters, and the cleaned ligation products (300–350 bp) were enriched by the PCR (15 cycles) with random primers (random hexamers), following by gel purification. Amplified libraries were checked by the Agilent 2100 Bioanalyzer (Agilent, Inc.). The experiments have three biological repetitions.

### RNA sequencing and data analysis

RNA sequencing was performed using Illumina HiSeqTM 4000 platform (Illumina, Inc.) for 150 bp paired-ends sequencing in Shanghai OE Biotech Co., Ltd. The samples were sequenced, and the raw data obtained in a FASTQ format were filtered with Cutadapt (v1.15) software to obtain clean data for further analysis. The filtered reads were aligned to the reference genome using TopHat2 upgraded HISAT2 software. Filtered reads were mapped to the reference genome using HISA T2 v2.0.5. HTSeq (0.9.1) was used to estimate the original expression level of the genes, and then the number of fragments per kilobase of transcript per million mapped reads (FPKM) was calculated to standardize the expression. Genes with |log2FoldChange| ≥ 1 and *P* value < 0.5 identified using DESeq (1.30.0) were considered as differentially expressed genes (DEGs).

The GO enrichment analysis was performed using the DEGs. The GO enrichment analysis was performed to assign possible functional categorization using AgriGO tool (http://systemsbiology.cau.edu.cn/agriGOv2/). The Kyoto Encyclopedia of Genes and Genomes (KEGG) pathway analysis of DEGs was performed using Cluster Profiler (3.4.4) software. Short Time-series Expression Miner (STEM) clustering was based on Ernst and Bar-Joseph [33].

### Quantitative RT-PCR analysis

To validate the RNA sequencing (RNA-Seq) results, ten genes were randomly selected for a quantitative reverse transcription PCR (qRT-PCR) analysis using a 2× SYBR GREEN Master Mix (Toyobo, Japan). Primers were designed using the NCBI database, and rice *Ubiquitin* was used as the reference gene. The thermal cycle of SYBR Green RT-PCR was as follows: 95 °C for 10 min and 40 cycles at 95 °C for 10 s and 60 °C for 30 s. The primers corresponding to the genes to be verified are listed in supplemental table S3. The comparative CT method (^ΔΔ^CT method) of quantification was used to quantify the relative expression of specific genes [34].

### Gas and liquid chromatography/mass spectrometry analysis

The samples that were analyzed for their transcriptome were also used for metabolome analysis, which was carried out by Shanghai Lu-Ming Biotech Co., Ltd. (Shanghai, China). Six biological replicates were taken in GC-MS and LC-MS for each time point. For GC-MS experiment, 100 mg of flash-frozen tissue samples were mixed with 60 μl of water containing ribitol as internal standard. The samples were mixed with 0.3 ml of methanol and 0.1 ml of chloroform and vortexed for 5 min followed by incubation at 70 ^o^C for 10 min. After centrifugation, supernatants were collected to be dried in a vacuum-dryer system. Following desiccation, each sample was incubated for 2 h at 37 ^o^C with 80 μl of methoxamine hydrochloride. Derivatization for gas-chromatography was performed with 1% trimethylchlorosilane (TMCS) in N-Methyl-N-(trimethylsilyl)-trifluoroacetamide (MSTFA) (100 μl) at 70◦C for 1 h. The derivatived samples were analyzed on an Agilent 7890B gas chromatography system coupled to an Agilent 5977AMSD system (Agilent Technologies Inc., CA, USA).

For LC-MS experiment, each sample was freeze-dried in a vacuum freeze-dryer and ground using a mixer mill with a zirconia bead for 2 min at 60 Hz. The 50 mg lyophilized samples were each dissolved in 1.2 ml 70% methanol. Following centrifugation at 13,400 g for 3 min, the extracts were filtered through a SCAA-1040.22-mm pore-size filter. ACQUITY UPLC I-Class system (Waters Corporation, Milford, USA) coupled with VION IMS QTOF Mass spectrometer( Waters Corporation, Milford, USA) was used to analyze the metabolic profiling in both ESI positive and ESI negative ion modes.

Differentially accumulated metabolites were determined based on the cut-off values of variable importance for projection ≥ 1 and absolute Log_2_FC ≥ 1.0. Identified metabolites were annotated according to the KEGG COMPOUND database (http://www.genome.jp/kegg/compound/), and then mapped using the KEGG PATHWAY database (http://www.genome.jp/kegg/pathway.html).

### Omics data merge and pathway analysis

Transcriptomics and metabolomics data for differentially expressed genes and regulated metabolite were integrated according to Subramanian et al. [35].

### Electronic supplementary material

Below is the link to the electronic supplementary material.

**Supplementary Material 1: Supplemental Fig. S1** The interaction networks among genes and metabolite. The light-yellow line means positive correlation, the light blue line represents negative correlation. **Supplemental Table S1** Parameters of quality control for the reads of each sample in the RNA-seq experiment. **Supplemental Table S2** Validation of the transcriptome data by qRT-PCR. **Supplemental Table S3** Primer information for qRT-PCR

**Supplementary Material 2: Supplemental data 1** Data of four DEMs in rice microspores under cold stress

## Data Availability

The datasets generated and analyzed during the current study are available in the National Center for Biotechnology Information. The raw data for RNA-seq can be downloaded at a https://www.ncbi.nlm.nih.gov/sra/PRJNA981281.

## Abbreviations

DEGs: Differentially expression genes
DEMs: Differentially expressed metabolites
DH: Doubled haploid
DPT: Days post treatment
Fluorescein Diacetate: FDA
FPKM: Fragments per kilo bases per million
GO: Gene ontology
KEGG: Kyoto Encyclopedia for Genes and Genomes
MDEC: Microspore-derived embryogenic calli
