# Supplementary material for: Transcriptomics integrated with metabolomics reveals the effect of cold stress on rice microspores
Source: BMC Plant Biol. 2023 Oct 27;23:521. doi: 10.1186/s12870-023-04530-2 (PMC10605337; doi:10.1186/s12870-023-04530-2)
Supplement: Supplementary file 1 — Supplementary Material 1: Supplemental Fig. S1 The interaction networks among genes and metabolite. The light-yellow line means positive correlation, the light blue line represents negative correlation. Supplemental Table S1 Parameters of quality control for the reads of each sample in the RNA-seq experiment. Supplemental Table S2 Validation of the transcriptome data by qRT-PCR. Supplemental Table S3 Primer information for qRT-PCR [file 12870_2023_4530_MOESM1_ESM.pdf]

Supplemental Fig. S1 The interaction networks among genes and metabolite. The light-yellow line means positive correlation, the light blue line represents negative correlation.

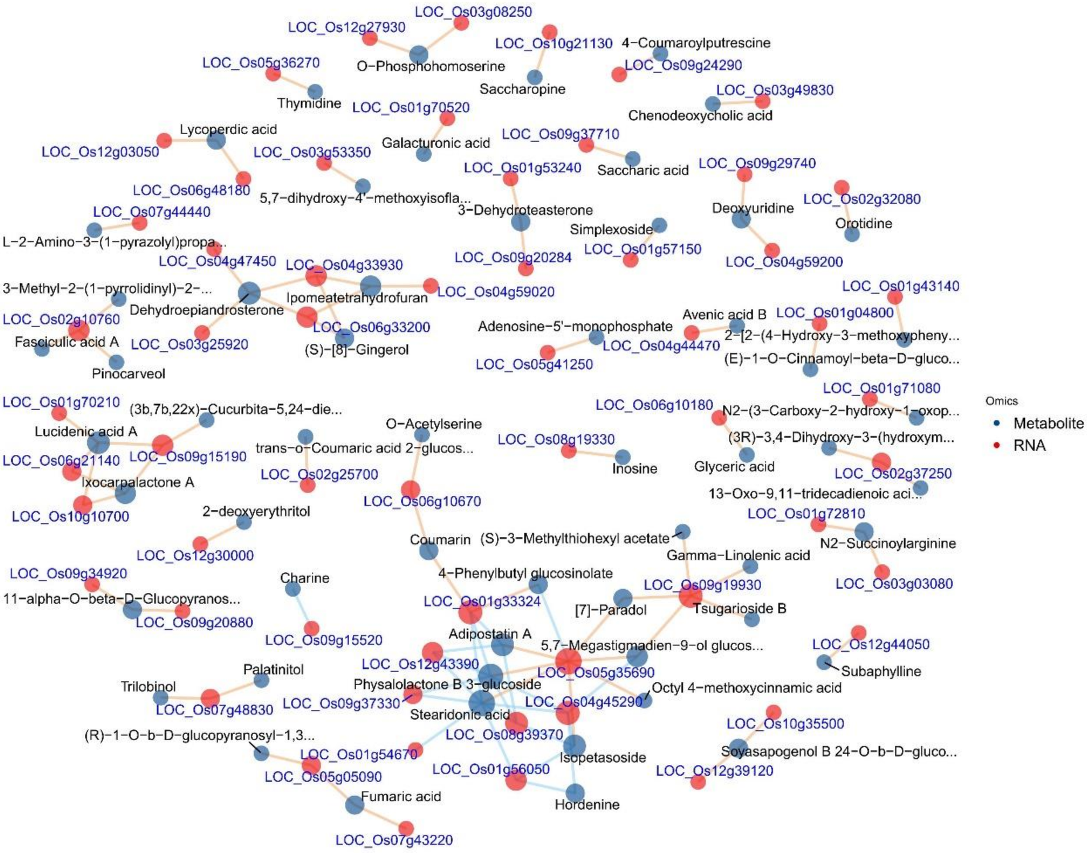

Supplemental Table S1 Parameters of quality control for the reads of each sample in the RNA-seq experiment.

Sample	RawReads	RawBases	CleanReads	CleanBases	ValidBases	Q30	GC
0 dpt_1	51.18M	7.68G	49.66M	6.71G	87.42%	91.66%	54.28%
0 dpt_2	49.77M	7.47G	48.15M	6.48G	86.76%	91.46%	53.57%
0 dpt_3	49.73M	7.46G	48.07M	6.51G	87.31%	91.35%	54.28%
3 dpt_1	47.99M	7.20G	46.55M	6.31G	87.61%	91.59%	53.88%
3 dpt_2	47.66M	7.15G	46.07M	6.12G	85.67%	91.43%	53.64%
3 dpt_3	47.84M	7.18G	46.33M	6.23G	86.81%	91.62%	53.86%
7 dpt_1	49.68M	7.45G	48.05M	6.50G	87.22%	91.46%	53.27%
7 dpt_2	47.95M	7.19G	46.35M	6.22G	86.51%	91.35%	53.77%
7 dpt_3	48.51M	7.28G	47.03M	6.35G	87.31%	91.76%	53.56%
10 dpt_1	48.89M	7.33G	47.22M	6.31G	86.00%	91.55%	53.83%
10 dpt_2	51.46M	7.72G	49.83M	6.73G	87.25%	91.52%	53.56%
10 dpt_3	49.83M	7.47G	48.13M	6.42G	85.95%	91.53%	53.65%

Supplemental Table S2 Validation of the transcriptome data by qRT-PCR.

		Fold change	
**Gene**		**qRT-PCR**	**RNA-Seq**
LOC_Os05g35690	3 dpt	-6.83949	-2.93083
	10 dpt	-63.8646	-42.2731
LOC_Os09g19930	3 dpt	-10.4816	-9.35958
	7 dpt	-16.0416	-12.0662
	10 dpt	-24.7004	-33.9024
LOC_Os04g45290	3 dpt	1.175388	6.175382
	7 dpt	1.962901	3.385084
	10 dpt	2.814846	2.072595
LOC_Os01g54670	7 dpt	8.258252	13.53716
	10 dpt	16.21246	19.49355
LOC_Os09g37330	3 dpt	2.052074	5.013401
	7 dpt	1.920633	9.411808
	10 dpt	13.60506	27.11591
LOC_Os12g43390	7 dpt	34.34457	31.84683
LOC_Os06g10670	7 dpt	2.227466	13.42553
	10 dpt	1.459181	22.44185
LOC_Os01g03390	7 dpt	5.444543	4.286993
	10 dpt	11.08021	8.595982
LOC_Os01g07890	7 dpt	3.62843	2.708293
	10 dpt	5.095853	3.833493
LOC_Os01g08170	3 dpt	1.19499	1.988663
	7 dpt	1.264315	1.03017

Supplemental Table S3 Primer information for qRT-PCR.

Gene	Q-PCR primer sequence
LOC_Os05g35690	F: GTCGAGACTACTGCTCCACA
	R: GTTGTGGGTGGTCATGTTGG
LOC_Os09g19930	F: TCACTCGTTGCGTCTATGGT
	R: CACACGGTGTTGCTGATCAA
LOC_Os04g45290	F: TGACATACTCAAGGGCTGGG
	R: CTCGATGTCCAGCTGTGTTG
LOC_Os01g54670	F: TCCATGGATGTCGGTCAAGT
	R: GCCCTTGTAGCTCCGTATCT
LOC_Os09g37330	F: TGGCGAACTCCTAAGGTTGT
	R: TGGCATTACGACAGAGCTCA
LOC_Os08g39370	F: TGCCTCATGTACTGCTCCAA
	R: TAACTGTGCCATCCCCAACT
LOC_Os01g56050	F: GCAGCCAAATTCTCGGTCAT
	R: TGAGGAATATGGTGGCTGCA
LOC_Os12g43390	F: TCTTCACCATCACCAACCAGT
	R: TCGTAGAAATCCAGCTGCTG
LOC_Os06g10670	F: TCACCAGCTTCTCCTACTGC
	R: CTGCGACGGGTTCTTGATG
LOC_Os01g03390	F: GCCATGACTACTTCCACCCT
	R: CTTCCACGGCTTCTTCTTCG
LOC_Os01g04670	F: CGACTACTACCGAGGCTACA
	R: GGATACCTCGAGATGACCGG
LOC_Os01g06836	F: CGACAATCAACTATCGGGCC
	R: TGGAAGAGAAGGGGACACTG
LOC_Os01g07890	F: GAGCTGAGACACTCCCGTAG
	R: ACAGAAGATGCATCCGGGAA
LOC_Os01g08170	F: TCAAGATCCTCATCCTCGCC
	R: GATGTACTCCTCCAGCGTCA
LOC_Os01g09100	F: CTTGTGCTTCCCTCCTCTCA
	R: ACGACACGCCATACTGATCA
Ubiquitin	F: AACCAGCTGAGGCCCAAGA
	R: ACGATTGATTTAACCAGTCCATGA
